# Obesity as pleiotropic risk state for metabolic and mental health throughout life

**DOI:** 10.1038/s41398-023-02447-w

**Published:** 2023-05-30

**Authors:** Michael Leutner, Elma Dervic, Luise Bellach, Peter Klimek, Stefan Thurner, Alexander Kautzky

**Affiliations:** 1 Department of Internal Medicine III, Clinical Division of Endocrinology and Metabolism, Medical University of Vienna, Waehringer Guertel 18–20, A-1090 Vienna, Austria; 2 Section for Science of Complex Systems, CeMSIIS, Medical University of Vienna, Spitalgasse 23, A-1090 Vienna, Austria; 3 Complexity Science Hub Vienna, Josefstaedter Straße 39, 1080 Vienna, Austria; 4 Department of Psychiatry and Psychotherapy, Medical University of Vienna, Waehringer Guertel 18-20, A-1090 Vienna, Austria

**Keywords:** Depression, Schizophrenia, Bipolar disorder

## Abstract

Obesity, a highly prevalent disorder and central diagnosis of the metabolic syndrome, is linked to mental health by clinical observations and biological pathways. Patients with a diagnosis of obesity may show long-lasting increases in risk for receiving psychiatric co-diagnoses. Austrian national registry data of inpatient services from 1997 to 2014 were analyzed to detect associations between a hospital diagnosis of obesity (ICD-10: E66) and disorders grouped by level-3 ICD-10 codes. Data were stratified by age decades and associations between each pair of diagnoses were computed with the Cochran-Mantel-Haenszel method, providing odds ratios (OR) and *p* values corrected for multiple testing. Further, directions of the associations were assessed by calculating time-order-ratios. Receiving a diagnosis of obesity significantly increased the odds for a large spectrum of psychiatric disorders across all age groups, including depression, psychosis-spectrum, anxiety, eating and personality disorders (all *p*_corr_ < 0.01, all OR > 1.5). For all co-diagnoses except for psychosis-spectrum, obesity was significantly more often the diagnosis received first. Further, significant sex differences were found for most disorders, with women showing increased risk for all disorders except schizophrenia and nicotine addiction. In addition to the well-recognized role in promoting disorders related to the metabolic syndrome and severe cardiometabolic sequalae, obesity commonly precedes severe mental health disorders. Risk is most pronounced in young age groups and particularly increased in female patients. Consequently, thorough screening for mental health problems in patients with obesity is urgently called for to allow prevention and facilitate adequate treatment.

## Background

Currently more than one in three people of the worldwide population is estimated to be overweight, underlining the urgency of obesity as a global health burden as prevalence rates have been steadily rising over the last decades [1]. In terms of abdominal fat obesity has a prominent role in the progression of metabolic disturbances of raised fasting glucose, high triglycerides and low high-density lipoproteins as well as high blood pressure, conceptually recognized as the metabolic syndrome. In addition, excessive body weight is clearly associated with psychiatric disorders and mental health [2]. While the collective prevalence and disease burden of mental health disorders is equally high and about 25% of the world population is expected to suffer from a psychiatric disorder, obesity is known to further ignite risk for and severity of mental illness [3]. In fact, two out of five people with overweight or obesity are diagnosed with a psychiatric disorder, especially mood, anxiety, psychosis spectrum (PSD) or eating disorders [4]. Patients with obesity have been shown to be at an increased risk for mood disorders such as major depressive disorder (MDD), bipolar disorder or dysthymia [5–8]. Interestingly, there seem to be discrepancies related to both sex and geographical region–women with obesity are more frequently diagnosed with depressive disorders compared to men [4, 9], likely due to cultural as well as biological reasons [10, 11]. Furthermore, patients with obesity in Europe are less likely to be diagnosed with depressive disorders than the United States of America [6]. Regarding the connection between obesity and eating disorders [12, 13], a similar gender gap with increased risk in women compared to men has been reported [4].

While the epidemiological association is clear, there is evidence for a bidirectional relationship between obesity and mental health that requires further research [5–7, 14]. However, the question of cause and consequence remains to be resolved [15–17]. The pattern of time-directionality between obesity and the development of psychiatric disorders such as MDD, anxiety disorders and PSD, is still a matter of debate that is fueled by the plethora of suggested biological and psycho-socio-economic links between these disorders. Next to reciprocal psychosocial impact of obesity on mental wellbeing and impaired mental health on lifestyle, biological links such as inflammation and cell stress [18, 19], but also iatrogenic effects such as the negative metabolic impact many psychopharmacological agents entail need to be addressed [20, 21].

In European countries such as Austria, but also most other part of the world, obesity is becoming more frequent and especially so in children and young adults. To apply preventive measures, it is of utmost importance to investigate diagnostic trajectories of obesity and common sequalae and comorbidities. Thus, the rationale for this study is to investigate across life decades the longitudinal association of obesity with mental health and somatic diagnoses. Population wide national health registry data is analyzed to assess bidirectional effects of obesity and comorbidities in form of risk increases over roughly one and a half decades.

## Methods

All analyses were performed within the national data registry of hospital stays in Austria across a period of 17 years, spanning from 1997 to 2014. Data covers ~45 million hospital stays of 9 million patients. Pseudonymized data were registered longitudinally for the Austrian population using inpatient services, including sex, age, admission and release dates, and all diagnoses received at each specific stay. Diagnoses are coded according to level-3 international statistical classification of diseases and related health problems (ICD-10) codes from chapters A to N. Thereby, the first level comprises the letter indicating the ICD chapter such as E for “endocrine, nutritional and metabolic diseases”. The second to third level comprise the two numbers indicating the specific disorders such as E66 for obesity. Names for all included ICD-10 chapters can be found in Supplemenary Figure 2. The remaining chapters coding diseases of pregnancy and childbirth (O), the perinatal period (P), congenital malformations (Q), abnormal clinical and laboratory findings (R), external factors influencing health and morbidity (S to Y) and factors influencing health status and contact with health services (Z) were not included.

We restricted our analysis to patients with hospital encounters between 2003 and 2014, excluding those treated as inpatients between 1997 to 2002. This was done following established standards to have comparable health status for the study population at the beginning of the observation period by means of not having required hospital services for five years [22]. An additional rationale for this procedure was that the ICD coding system was changed in Austria in the early 2000s and including data gathered before 2002 would impede comparability of diagnoses. A graphical overview of the cohort selection process is provided in Supplementary Figure 1.

Patients were stratified by having a diagnosis of obesity according to ICD-10 criteria. Next, comorbidities of obesity were assessed across chapters A to N of the ICD-10 catalog. Considering that comorbidities are strongly age dependent, we analyzed significant comorbidities in 7 age groups defined by the decade of life (10–19, 20–29, and so on). Each age group stratum was further stratified based on 2-year time windows (2003–2004, 2005–2006, and so on). Within each age group and each 2-year stratum, for each pair of diagnoses a contingency table was built that was used to calculate a weighted average of the estimates of the risk ratios, odds ratios (OR) and *p* values using the Cochran-Mantel-Haenszel method [23]. Stratified analysis was performed to adjust for confounding variables (absolute age within age group, time period). Associations with comorbidities with less than 100 occurrences were deleted as a quality control measure. Filtered comorbidities with an odds ratio (OR) higher than 1.5 and a *p* value smaller than 0.01 after Bonferroni multiple testing correction are reported as significant comorbidities, with special focus on codes 10–69 of the F chapter, describing adult psychiatric disorders. Results are reported for sufficiently frequent and significant comorbidities of obesity in the form of percentages that give the relative frequency of these diagnoses in patients with obesity as well as OR with 95% confidence intervals (CI).

In the next step of analysis, we further stratified the study sample by sex to assess whether women and men with and without obesity show different patterns of associations. Calculations were performed similar as described above for the total sample. Within patients with obesity, OR with CI were computed for sex-specific risk of receiving diagnoses of psychiatric comorbidities.

Finally, for sensitivity analysis patients with a diagnosis of either diabetes (E11), hypertension (I10) or coronary artery disease (I25) were excluded and links with obesity were calculated as described above. We assessed specifically whether links to depression (F32 or F33) are still present after exclusion of these known risk factors.

### Time directionality

We calculated the difference in time between each diagnosis pair for every patient in the studied period. Patients are organized into four groups: (1) Both diagnoses are diagnosed during the same hospital stay, or the time difference between diagnoses was (2) less than ~1 year (360 days), (3) longer than 1 year, and less than 3 years, or (4) greater than 3 years. We calculated the time-order ratio (TOR) as the number of patients where the direction is from diagnosis A to diagnosis B, divided by the number of patients with direction B to A in each defined group. TOR (A → B) of <1 indicates a diagnosis B tends to occur before a diagnosis A, and TOR (A → B) of >1 indicates a tendency of opposite direction. In this analysis, obesity was always used as disorder A and each linked comorbidity as a disorder B. Consequently, a TOR of 2 indicates twice as many people diagnosed first with obesity than those diagnosed first with the respective comorbidity. Similarly, a TOR of 0.5 indicates twice as many patients diagnosed first with the comorbidity than those diagnosed first with obesity. Assuming that both counts stem from a binomial distribution with equal success probability, we tested the null hypothesis that N (A → B) = N (B → A) to see if TOR (A → B) is significantly different from 1 [24].

## Results

A total of 3,006,526 patients without obesity (50% female, mean age 44.41 ± 18.2 years) and 161,185 patients with obesity (51% female, mean age 53.1 ± 16.6 years) were analyzed. The average number of diagnoses (10.3 vs 4.0), hospital stays (5.7 vs 2.8) as well as hospital days (40.9 vs 15.8) were higher in patients with a diagnosis of obesity. A summary of sample characteristics and average co-diagnosis rates for selected comorbidities is presented in Table 1.

**Table 1:** Sample characteristics for patients with and without a diagnosis of obesity, respectively for the total samples and male and female patients.

	Obesity (ICD-10: E66)			No obesity		
	Total	Female	Male	Total	Female	Male
Number of subjects	163,185	82,846	80,339	3,006,526	1,510,543	1,495,983
Age (mean ± SD)	53.11 ± 16.59	52.62 ± 17.39	53.61 ± 15.7	44.41 ± 18.2	43.89 ± 18.18	44.92 ± 18.21
Hospital Stays (mean ± SD)	5.68 ± 7.14	5.58 ± 6.88	5.78 ± 7.40	2.84 ± 4.68	2.80 ± 4.56	2.87 ± 4.80
Hospital Days (mean ± SD)	40.85 ± 66.54	41.39 ± 65.75	40.29 ± 67.33	15.78 ± 43.40	14.92 ± 41.54	16.65 ± 45.19
Number of diagnoses (mean ± SD)	10.31 ± 7.96	10.08 ± 7.98	10.54 ± 7.93	3.95 ± 4.41	3.82 ± 4.22	4.08 ± 4.58
Co-diagnoses *(%)*						
Diabetes mellitus type 2 (E11)	24.37	21.63	27.20	3.72	2.76	4.68
Lipidemia (E78)	35.04	29.17	41.08	7.71	6.10	9.34
Hypertonia (I10)	59.78	54.57	65.16	14.89	13.01	16.79
Coronary artery disease (I25)	18.29	12.50	24.27	4.45	2.58	6.33
Atrial fibrillation (I48)	10.48	8.49	12.53	2.76	2.05	3.47
COPD (J44)	10.80	8.35	13.32	2.45	1.71	3.21
Insomnia (G47)	12.35	5.84	19.06	1.51	0.75	2.27
Nicotine use disorder (F17)	12.56	8.58	16.66	3.73	2.63	4.83
Schizophrenia (F20)	0.94	1.02	0.87	0.54	0.41	0.67
Schizoaffective Disorderd (F25	0.63	0.82	0.43	0.22	0.24	0.2
Bipolar disorder (F31)	0.73	0.88	0.57	0.36	0.39	0.33
Depressive episode (F32)	10.01	13.3	6.61	4.01	4.80	3.21
Recurrent depression (F33)	3.53	4.71	2.32	1.43	1.74	1.12
Dysthymia (F34)	0.73	1.00	0.45	0.27	0.33	0.21
Anxiety disorder (F41)	2.71	3.44	1.97	1.29	1.61	0.97
Somatization disorder (F45)	1.66	2.22	1.08	0.87	1.11	0.63
Eating disorder (F50)	0.52	0.79	0.23	0.29	0.51	0.07
Personality disorder (F60)	1.09	1.49	0.68	0.70	0.76	0.65

*COPD* chronic obstructive pulmonary disease, *SD* standard deviation.

For selected ICD-10 codes, percentages of patients having received the respective diagnosis are presented.

Specific diagnoses most commonly co-occurring with obesity were disorders related to the metabolic syndrome, such as diabetes mellitus type 2 (E11, 23.97%), arterial dyslipidemia (E78, 34.5%) and hypertension (I10, 58.84%). While the metabolic syndrome is not recognized by a specific ICD-10 code and thus could not be targeted directly, these disorders resemble most closely the required metabolic disturbances. Similar patterns were recognized for other well-established sequalae such as coronary artery disease (I25, 17.99%), arrhythmia (I48, 10.3%) and chronic obstructive pulmonary disease (J44, 10.68%). Obesity was further linked to a co-diagnosis of insomnia (G47, 12.18%) across all age groups. For all these comorbidities, diagnosis rates were increasing with age and peaked in old age.

### Psychiatric diagnoses in patients with and without obesity

The highest concentration of links from a diagnosis of obesity to other diagnoses grouped by ICD-10 chapters A to N was found within the F chapter, especially for young patients up to 30 years of age (Supplementary Figure 2). Across the total sample, risk increases in patients with a diagnosis of obesity compared to those without were observed for nicotine use disorder (F17, OR 3.71 [CI 3.65–3.77]), schizophrenia (F20, OR 1.75 [CI 1.66–1.84]), schizoaffective disorder (F25, OR 2.88 [CI 2.69–3.07]), bipolar disorder (F31, OR 2.03 [CI 1.92–2.16]), depressive episodes (F32, OR 2.52 [CI 2.45–2.59]), recurrent depression (F33, OR 2.65 [CI 2.61–2.70]), dysthymia (F34, OR 2.72 [CI 2.56–2.89]), anxiety disorders (F40, OR 2.13 [CI 2.07–2.20]), somatization disorders (F45, OR 1.92 [CI 1.85–2.00]), eating disorders (F50, OR 1.80 [CI 1.68–1.93]), as well as personality disorders (F60, OR 1.56 [CI 1.49–1.64]).

Around 60% of co-diagnoses with nicotine use disorder were received within the same hospital stay as the diagnosis of obesity. For the remaining patients with both disorders, obesity was significantly more often the first diagnosis and even more so in women with a highest TOR of approximately 1.5 when diagnoses were received more than three years apart.

Around a quarter of patients received a diagnosis of obesity and respectively F32 or F33 within the same hospital stay, while the remaining three quarters distributed evenly across the observation period. Regarding both single and recurrent episodes, obesity was significantly more often the diagnosis received first. Highest TOR of approximately 2 were observed when time between diagnoses exceeded three years.

Both anxiety and somatization disorders were diagnosed within the same hospital stay in approximately a third of patients. For the remaining patients with both diagnoses, anxiety disorders showed TORs between 1.5 and 2 and thus obesity was significantly more often the disorder diagnosed first. For somatization disorders highest TOR of 2 were observed in women diagnosed with both disorders within a year. In women, obesity was significantly more often the first diagnosis when three or more years went by between diagnoses.

Around a third of patients with obesity and a diagnosis of schizoaffective disorder or schizophrenia received both diagnosis within the same hospital stay. For the remaining patients, contrary to all other psychiatric co-diagnoses, the psychosis-spectrum disorders were suggested to be the diagnosis received first. TOR decreased with time between diagnoses and reached a low point of ~0.5 for schizophrenia in men when three years were exceeded.

No significant time pattern of co-diagnosis with eating or personality disorders was detected.

**Fig. 1:**
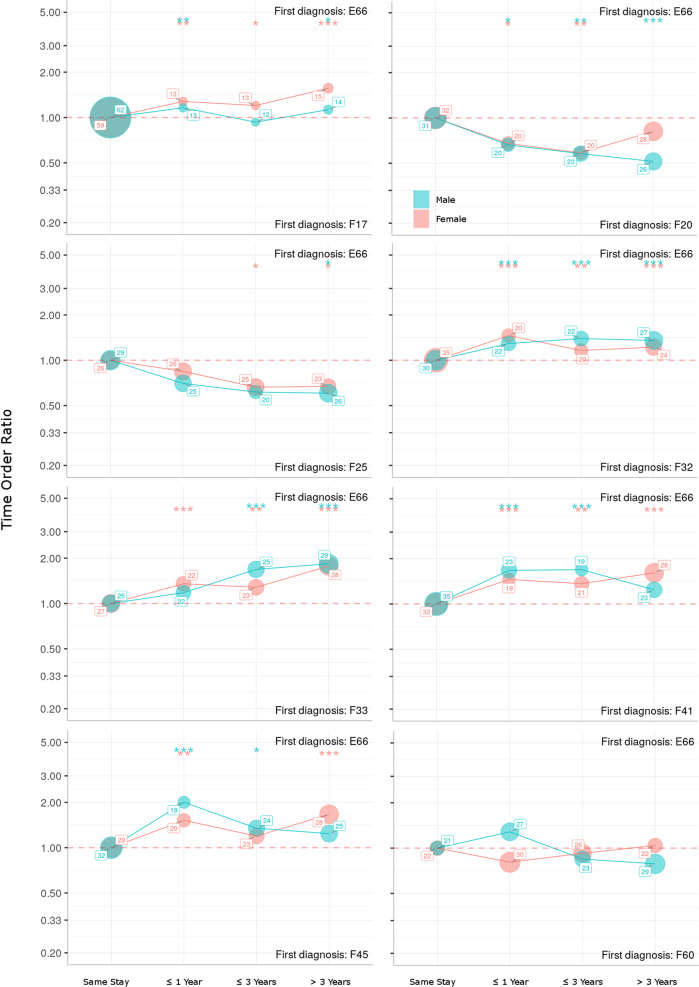
Time-ordering ratios for patients with a diagnosis of obesity and a respective psychiatric co-diagnosis grouped by two-digit ICD-10 F codes, stratified by sex. Percentages of patients who receive both diagnosis at the same hospital stay, within a year, within 3 years and with a time gap of >3 years are presented for each sex. Lines in the upper half of the diagram indicate obesity to be the first diagnosis received while lines in the lower half indicate the respective psychiatric disorder to be the first diagnosis received. For example, when time between diagnoses exceeded three years a ratio of ~2 was observed for recurrent depressive disorder (F33), indicating that there were twice as many people diagnosed first with obesity than those diagnosed first with depression. Time-order-ratios tested for significance (*p < 0.05, **p < 0.01, ***p < 0.001).

For a summary of time-order relations, stratifying patients with obesity by time between receiving co-diagnoses, please see Fig. 1.

### Stratification by age

**Fig. 2:**
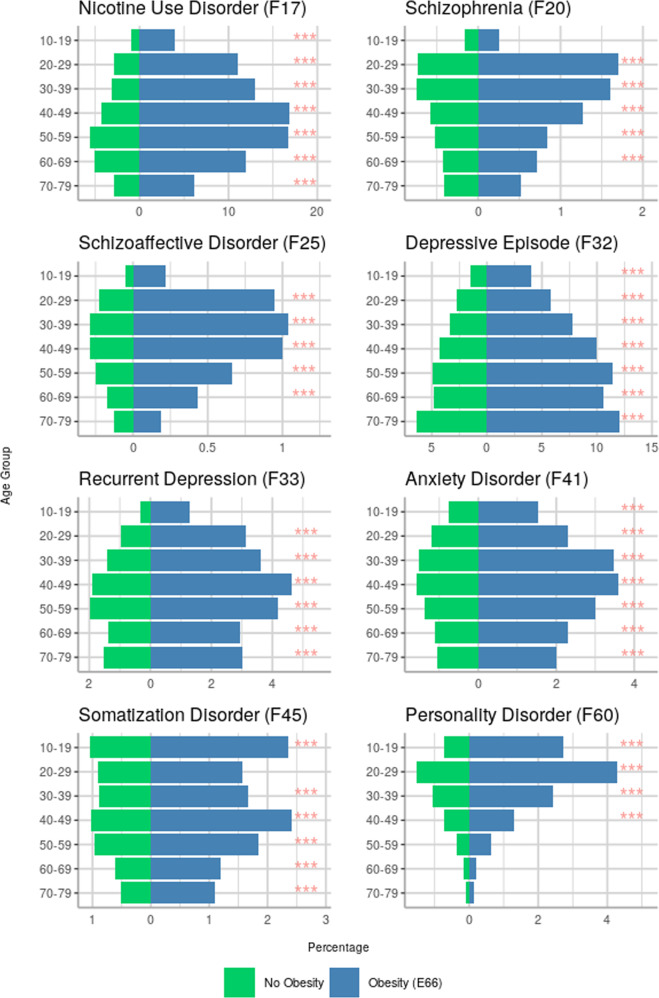
Percentages of patients with and without obesity with a diagnosed psychiatric disorder, grouped by two-digit ICD-10 F codes and by age decade. *p* values are presented by levels of significance (*** *p*_corr_ < 0.001).

Results of risk increases in patients with obesity for psychiatric comorbidities stratified by age are summarized in Table 2. The most important associations are depicted in Fig. 2, presenting percentages of patients with and without obesity respectively for each psychiatric disorder with corresponding OR.

**Table 2:** Odds ratios (OR) with 95% confidence intervals (CI) for patients with obesity to receive an additional mental health diagnosis stratified by decade of life.

ICD-10	Age	OR [CI]	*p*-value	Diagnosis rate (%) in patients with obesity
F17 Nicotine use	10–19	4.95 [4.34–5.64]	<0.01	3.98
	20–29	5.58 [5.21–5.97]		10.99
	30–39	5.45 [5.19–5.72]		13.01
	40–49	4.88 [4.72–5.04]		16.87
	50–59	3.61 [3.50–3.72]		16.71
	60–69	2.79 [2.69–2.90]		11.9
	70–79	2.62 [2.46–2.79]		6.14
F20 Schizophrenia	20–29	1.88 [1.62–2.17]	<0.01	1.7
	30–39	1.84 [1.62–2.09]		1.6
	40–49	1.75 [1.56–1.96]		1.27
	50–59	1.53 [1.36–1.73]		0.84
	60–69	1.60 [1.39–1.84]		0.72
F25 Schizoaffective dis.	20–29	3.39 [2.77–4.15]		0.95
	30–39	3.01 [2.58–3.51]		1.04
	40–49	2.86 [2.52–3.25]	<0.01	1
	50–59	2.59 [2.25–2.98]		0.66
	60–69	2.02 [1.65–2.47]		0.43
F31 Bipolar dis.	10–19	<100 patients		0.45
	20–29			0.83
	30–39	1.63 [1.37–1.93]	<0.01	1
	40–49	1.65 [1.45–1.88]		1.04
	50–59	1.59 [1.39–1.81]		0.78
	60–69	2.01 [1.72–2.35]		0.64
	70–79	<100 patients		0.32
F32 Depressive episode	10–19	2.13 [1.89–2.40]	<0.01	4
	20–29	1.61 [1.46–1.78]		5.78
	30–39	1.96 [1.84–2.09]		7.75
	40–49	2.10 [2.01–2.20]		10
	50–59	2.35 [2.26–2.43]		11.44
	60–69	2.19 [2.10–2.28]		10.56
	70–79	2.04 [1.95–2.13]		12.06
F33 Recurrent depression	10–19	<100 patients		1.27
	20–29	2.24 [1.96–2.56]	<0.01	3.12
	30–39	2.02 [1.84–2.21]		3.63
	40–49	1.82 [1.70–1.94]		4.62
	50–59	1.85 [1.74–1.96]		4.18
	60–69	1.88 [1.74–2.04]		2.95
	70–79	1.83 [1.67–2.01]		3.03
F34 Dysthymia	20–29	<100 patients		0.44
	30–39			0.46
	40–49	1.94 [1.62–2.31]	<0.01	0.73
	50–59	2.18 [1.89–2.52]		0.81
	60–69	2.39 [2.06–2.78]		0.82
	70–79	2.00 [1.68–2.37]		0.9
F41 Anxiety dis.	10–19	1.93 [1.59–2.35]	<0.01	1.53
	20–29	1.75 [1.52–2.02]		2.29
	30–39	1.84 [1.67–2.03]		3.48
	40–49	1.97 [1.82–2.12]		3.59
	50–59	1.97 [1.83–2.12]		3
	60–69	2.16 [1.98–2.36]		2.3
	70–79	1.97 [1.76–2.21]		2.01
F45 Somatization dis.	10–19	2.04 [1.74–2.40]	<0.01	2.36
	30–39	1.53 [1.32–1.77]		1.66
	40–49	1.88 [1.71–2.07]		2.41
	50–59	1.78 [1.62–1.95]		1.84
	60–69	1.78 [1.56–2.03]		1.19
	70–79	1.90 [1.61–2.25]		1.09
F50 Eating dis.	10–19	2.24 [1.89–2.65]	<0.01	1.89
	20–29	1.98 [1.65–2.37]		1.65
	30–39	2.81 [2.32–3.40]		0.97
	40–49	4.89 [4.13–5.80]		0.62
	50–59	<100 patients		0.31
	60–69			0.13
F60 Personality dis.	10–19	2.71 [2.34–3.14]	<0.01	2.73
	20–29	2.18 [1.95–2.42]		4.27
	30–39	1.89 [1.69–2.12]		2.42
	40–49	1.50 [1.33–1.70]		1.31
	60–69	<100 patients		0.21

All listed associations remained significant after correction for multiple testing. Diagnoses that were present in fewer than 100 patients with obesity within a specific age decade were not analyzed.

Nicotine use disorder was linked to obesity across all age groups. The lowest fraction of 3.98% but also the highest increase in risk with an OR of 4.95 (CI 4.34–5.64) was found within those receiving a diagnosis of obesity between 10–19 years of age, compared to highest occurrence rates of almost 17% in middle aged patients of 40–59 life years.

For depressive episodes, a linear increase of co-diagnosis rates with obesity with age was observed (4.0% in age group 10–19 vs 12.1% in age group 70–79). Risk increases in patients with obesity ranged from OR 1.65 to 2.35. Rates of recurrent depression were highest in patients receiving a diagnosis of obesity between 40–59 years of age (4.62%). Risk increases were most pronounced in young patients (20-29 years: OR 2.24) and decreasing with age (OR < 1.9 in patients aged 40+).

Co-diagnosis rates of obesity and anxiety disorders ranged from 1.53% in youngest patients to rates above 3% in patients aged 30–59. Risk was about doubled in patients with obesity in all age groups with ORs ranging from 1.75 to 2.16. Concerning somatization disorders, highest increases in risk (OR 2.04) as well as highest co-diagnoses rates (2.36%) were observed in the youngest group aged 10–19.

Further, links between a diagnosis of obesity and schizophrenia as well as schizoaffective disorders were identified in patients aged 20–69 years. Rates of co-diagnosis and risk were decreasing with age for both disorders, with highest ORs of respectively 1.88 for F20 and 3.39 for F25 observed in young patients aged 20–29 years.

Regarding eating disorders, co-diagnosis rates were decreasing with age, but risk increases were more pronounced in older age groups with an OR of 4.89 in patients with obesity aged 40–49.

Finally, obesity co-occurring with personality disorders peaked within patients of 20–29 years of age (4.27%) and sharply dropped in older patients. Risk increases were highest in youngest patients aged 10–19 years (OR 2.71) and similarly decreasing with age.

Regarding sensitivity analysis, links from obesity to depressive episodes and recurrent depression were also observed after exclusion of patients with either diabetes, arterial hypertension or coronary artery disease. Significant associations (all *p*_corr_ < 0.01) were computed across all age groups except youngest patients aged 10–19 years and present in both females (ORs 1.7–2.3) and males (ORs 1.3–1.6).

### Sex differences among psychiatric comorbidities

**Fig. 3:**
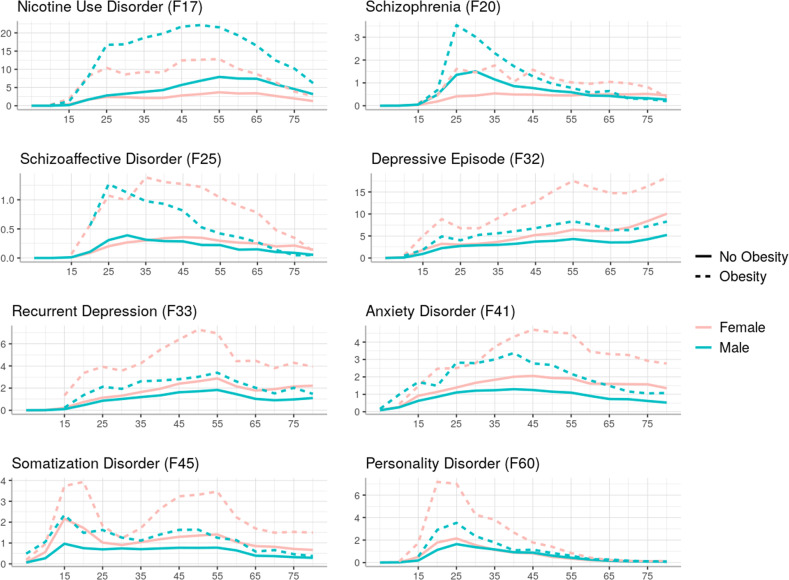
Diagnosis rates for common psychiatric comorbidities stratified by presence and absence of a diagnosis of obesity (ICD-10: E66) and by sex. Gender gaps towards male overrepresentation are seen in schizophrenia and nicotine use disorders, while all other comorbidities were showed increased risk in females. Gender gaps further widened in the presence of a diagnosis of obesity.

Stratifying the sample by sex revealed a gender gap for all psychiatric diagnoses linked to obesity. As depicted in Fig. 3, sex differences already present for patients without obesity were boosted in patients with a diagnosis of obesity. For depressive and somatization disorders, consistent overrepresentation of female patients with obesity with on average doubled risk was observed throughout age groups. While female sex was also more common among patients with obesity with anxiety disorders, significant risk increases were only observed in patients beyond 40 years of age (respective ORs 1.61–2.89). For young patients with obesity aged 10–19 years increased risk for diagnosis of personality disorder was observed in women compared to men (OR 4).

**Fig. 4:**
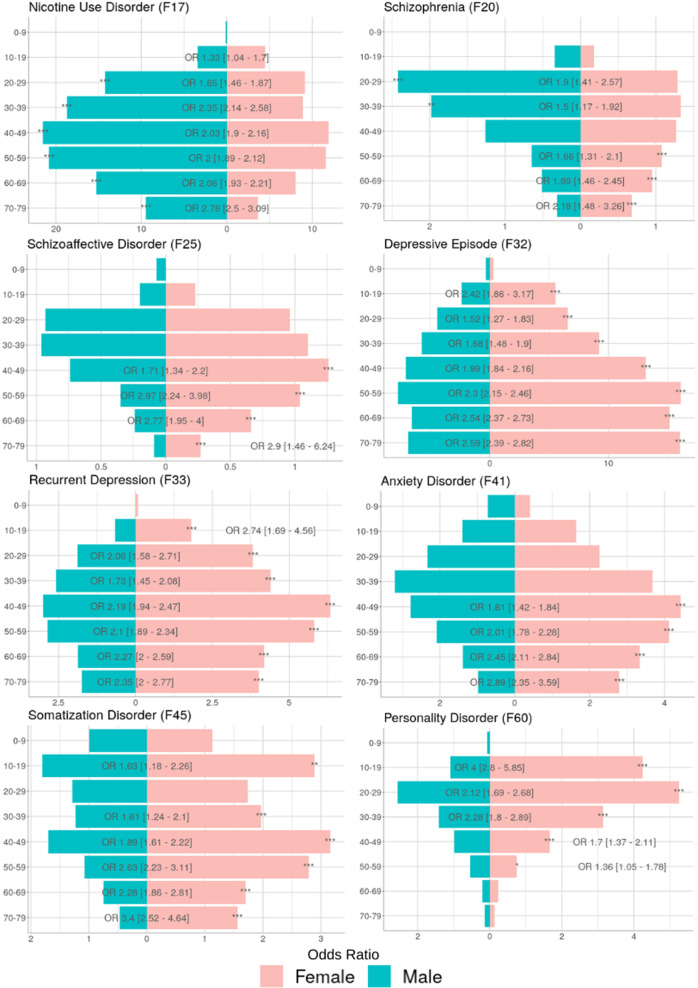
Stratification by sex for patients with a diagnosis of obesity and a respective psychiatric co-diagnosis grouped by two-digit ICD-10 F codes. Odds ratios (OR) with confidence intervals are presenting the risk of being female or male when a respective psychiatric co-diagnosis is received for each age group. Corrected *p* values are presented by levels of significance (*p < 0.05, **p < 0.01, ***p < 0.001).

For nicotine addiction as well as psychosis spectrum disorders, different risk increases by sex were observed dependent on age groups. Nicotine addiction was associated with male sex in all age groups (respective ORs 1.65–2.78 for males compared to females) except in the youngest patients with obesity 10–19 years of age (OR 1.33 for females compared to males). For schizophrenia and schizoaffective disorder, increased risk was observed in men compared to women in younger patients with obesity aged 20–39 (ORs 1.5–1.9) but in women compared to men in older age groups 40–79 (ORs 1.34–2.24). A summary of risk dependent on sex can be found in Fig. 4.

## Discussion

Exploiting population-wide Austrian registry data, at each decade of adult life obesity was identified as a relevant risk factor for receiving additional mental health diagnoses, underlining the importance of obesity as a pleiotropic promotor of health problems.

Among singular ICD-10 diagnoses, links to diabetes mellitus, arterial hypertension and dyslipidemia emerged as the most robust associations with obesity. This further underlines the well-established role of obesity as a driving factor in metabolic dysregulation, as it constitutes the most important risk factor for diabetes mellitus type 2 and in terms of abdominal obesity places central in the concept of metabolic syndrome [25, 26]. Consistently, with increasing age also a variety of known sequelae of the metabolic syndrome such as chronic ischemic heart disease [27], arrhythmia [28] and chronic obstructive pulmonary disorders were identified [29]. The highest risk increase in young patient groups emphasizes the decisive role of obesity for early development of diabetes mellitus and other disorders usually associated with aging [15]. These findings are well in line with clinical observations and established etiopathological models, thus raising confidence in the direction and validity of our registry-based results.

Comorbidity with obesity particularly manifests in mental health as links to the F chapter were most abundant across the whole ICD-10 catalog in young age groups. Expectedly, the average number of diagnoses was higher in older age groups compared to younger patients, bringing along also more links between obesity and psychiatric comorbidities. While the majority of diagnoses were received by older age groups, the risk for psychiatric diagnoses in patients with obesity compared to those without was most increased in earlier decades of life. Occurrence of depressive, anxiety and schizoaffective disorders as well as schizophrenia was more common in patients with obesity across all age groups. With less consistency, risk increases were also confirmed for bipolar disorder, dysthymia and eating disorders. These diagnoses were previously demonstrated to be more frequent in patients with obesity [7]. Our data thus support the bidirectional links between obesity and mental health and show that psychiatric comorbidities are commonly manifested within a few years.

Abundant evidence linking mental health disorders to obesity has already been presented [30, 31]. Among biological pathways linking obesity to mental health, increased neuroinflammation due to cytokine production in adipocytes may be most prominent [32, 33]. Further links include shared nutritional risk factors as high fat Western diet was demonstrated to increase inflammation and impact negatively on neurotrophic factors and the gut microbiome that is implicated both in obesity and mental health [19]. Concerning psychosocial links between physical and mental health, severe mental health disorders interfere with the ability to enjoy and pursue one´s interests and consequently impact daily living and quality of life fundamentally [34]. Compromises to healthy lifestyle may lead to weight gain due to reduced physical activity [35]. Here, obesity was identified as the diagnosis commonly preceding psychiatric comorbidities, even though we cannot rule out that a relevant portion of patients had received a diagnosis of obesity first because an underlying psychiatric disorder was not adequately diagnosed at that time. For example, manifest psychosis is often preceded by years of prodromal states with mostly unspecific psychiatric symptoms that may be attributed wrongly [36]. In addition to the shared biological substrates such as inflammation, progression from obesity to mental health disorders may also be mediated by disease burden of chronic metabolic disorders such as diabetes and other disorders relevant to the metabolic syndrome. In fact, we observed high rates of diabetes mellitus among patients with obesity with a co-diagnosis of major depression (F32/33; 33.26% among females, 39.48% among males) and psychosis spectrum disorders (F20/25; 32.03% among women, 31.39% among men). Specific syndromes such as diabetes distress are established in clinical practice but not depicted in diagnostic manuals [16]. As they mostly feature symptoms of anxiety and mood disorders, disease-specific syndromes related to chronic disorders such as diabetes may partly be responsible for the links to depression and anxiety disorders observed here. However, associations with depression remained significant after excluding patients with known somatic risk factors diabetes, arterial hypertension and coronary artery disease. Our findings are thus in line with previous reports of increased occurrence of depression in obesity also when controlling for other chronic disorders as well as sociodemographic characteristics [37]. Interestingly, depressive subtypes may associate differently with obesity as indicated by a recent meta-analysis linking obesity specifically to atypical depression, that was associated with more than doubled BMI compared to other subtypes [38]. Considering that depressive subtypes are not coded by ICD-10, we cannot follow up on these findings with the current analysis.

Another confounding factor are iatrogenic effects mediated by antidepressant and even more so antipsychotic medication [20, 35]. While we cannot account for medication effects in this data context, co-diagnoses observed for mood and anxiety disorders indicate a pattern of obesity being diagnosed first and putatively before neuropsychiatric medication is prescribed. On the contrary, schizophrenia was frequently anteceding obesity especially in younger patients. Consequently, orexigenic and diabetogenic effects of antipsychotics may be responsible for this link. On the other hand, clinical data suggested impaired glucose trafficking in high-risk individuals for psychosis-spectrum as well as affective disorders even before first episodes occur and any medication was prescribed. The observed pattern of obesity anteceding depressive and anxiety disorders is in line with a proposed transdiagnostic link to glucose dysregulation in prodromal and early onset mental health disorders [39].

Furthermore, we observed substantial sex differences for psychiatric disorders linked to obesity. The higher number of women with depression and anxiety disorders is a well-known finding with about doubled prevalence rates in women found worldwide [40, 41]. Similarly, higher rates of psychosis spectrum and substance use disorders in men were consistently reported [42, 43]. Biologic factors such as sex hormones as well as psychosocial burden such as a higher risk for adverse life events and societal disparities contribute to this gender gap in disease prevalence, that can however not be addressed by the current results. Importantly, these well-known sex differences in mental health disorders are here shown to be magnified in the presence of obesity. In the case of depression, in presence of obesity women showed tripled rates of diagnosed depressive episodes (13.3% in patients with vs 4.8% in patients without obesity) compared to men with doubled rates (6.61% in patients with vs 3.21% in patients without obesity). Regarding psychosis spectrum disorders, in the presence of obesity the male overrepresentation typically observed in epidemiological studies only occurred in the first half of life with women matching male diagnoses rates around the age of 40 and surpassing them beyond this age. This may be owed to a reduced protective effect of female sex hormones against psychosis onset in women with obesity, potentially mediated by altered hypothalamus-pituitary-adreno signaling that accelerates the rise in psychosis spectrum disorders commonly observed in perimenopausal states [44, 45].

Regarding limitations, this is a register-based analyses of hospital diagnoses. Previous results on mental health and obesity were inconsistent and some studies suggested effects to be moderated by other somatic conditions and socioeconomic status [46]. Psychosociodemographic characteristics were not available for this sample, nor could family history of metabolic or mental health disorders or drug prescription data be implemented. The lack of more detailed characterization of the patient cohorts eliminated options to analyze clinical features such as BMI and abdominal obesity, inflammation or depressive subtypes and symptom severity. While the longitudinal nature of the data pool allowed estimation of time-order ratios, the intervals considered here may have been too small to disentangle relevant directionalities. Especially diagnoses of obesity received early in life may bear risk for subsequent development of psychiatric disorders. There are established links between obesity and mental health disorders predominantly diagnosed in childhood and adolescence such as conduct disorder or attention deficit hyperactivity disorder [47], but also childhood depression and anxiety disorders [48] that showed ramifications into adult mental health [49]. Unfortunately, as only data of the time window from 2002 to 2014 could be analyzed, samples of specific diagnoses pairs were too small to consider long term effects across age decades. For the same reason, we chose time windows for TOR between diagnosis pairs “up to three years” and “beyond 3 years”. An alternative selection of time windows up to five years and beyond for TOR produced similar results. Considering that for several diagnoses highest TOR were observed when the gap between diagnoses was largest, we cannot rule out that longer time intervals are more suitable for future analyses.

Thus, reflecting on the limitations we cannot clarify underlying mechanisms of the association between obesity and mental health disorders. Nevertheless, our results clearly underline the connection between obesity a diagnosis of depressive, anxiety and psychosis-spectrum disorders starting from young adult until old age.

Furthermore, lower numbers of diagnoses were observed compared to established point prevalence in western countries, such as around 5% for men and 10% for women for MDD [50]. Similarly, the average rate of obesity of 4.63% was below estimates of around 11% for the Austrian population. While comorbid MDD was shown to be quadrupled in patients with severe obesity indicated by BMI > 35 [51–53], here we found lower rates of 11.37% of patients with and 4.27% patients without obesity to be diagnosed with a depressive episode or recurrent depression. On one hand, disorders must be expected to be underdiagnosed across all entities due to known shortcomings of registry data. On the other hand, some bias may stem from disproportionate diagnostic gaps that may be especially relevant for psychiatric disorders. Receiving the correct diagnosis and adequate pharmacological care is known to oftentimes follow years of delay in mental health. Furthermore, psychiatric diagnoses are mostly established in specialized centers while they are often improperly recognized in non-psychiatric inpatient care. In contrast, disorders that share a clinically recognized and therapeutically potent connection with obesity such as diabetes, hypertension and heart disease, can be expected to be routinely coded and better represented in a registry of inpatient-diagnoses. Along these lines, the fact that patients with obesity had roughly doubled average numbers of diagnoses and inpatient stays not only supports a pleiotropic role of obesity in disease progression, but also a higher probability of these patients being correctly diagnosed with all relevant comorbidities compared to patients with less exposure to the health care system. In any way, our results advocate to always include a diagnosis of obesity for patients fulfilling BMI criteria. While on one hand patients can be stigmatized by a diagnosis of obesity and opinions not to regard obesity as a disease still exist [54], the clear link to a plethora of high impact chronic diseases calls for early recognition to facilitate treatment and prevention measures [55].

Irrespective of the underlying pathways and expectedly lower numbers compared to natural studies, our study shows several strengths. First, exploiting Austrian national registry data our results can be regarded representative for the Austrian health care system. Second, by including the whole range of the ICD-10 catalog for analysis we identified psychiatric comorbidities to be among the most abundant associations with obesity. Third, by stratifying by age and sex, important influencing factors are accounted for. The scope of the findings clearly calls for better awareness of mental health disorders in patients with obesity. Based on the well-established etiopathological overlap between mental health, obesity and endocrine health, some guidelines have already incorporated recommendations addressing mental comorbidity [56–58]. However, the majority of these guidelines are targeting management of obesity in patients with diagnosed mental health disorders or vulnerable groups with obesity such as patients eligible for bariatric surgery [2]. Some guidelines also call for regular screening for mental health disorders, specifically attention deficit disorder, depression, anxiety and substance use, however, lacking standardized recommendations for screening tools and timelines. Based on our results, routine screenings for depressive episodes, anxiety and somatization, psychosis-spectrum such as schizophrenia and schizoaffective as well as personality disorders are called for whenever establishing a diagnosis of obesity. Importantly, increases in risk for being diagnosed with a mental health disorder following a diagnosis of obesity were shown to be long lasting and advise clinicians to keep checking for relevant symptoms to allow timely and adequate treatment.

## Supplementary information

Supplemental Material

## Data Availability

The analysis is based on secondary use of a research database of medical claims records, which is safeguarded and maintained by the Federal Ministry of Health. This is a consolidated research database that is only accessible for selected research partners under a strict data protection policy. Use of the data takes place in agreement and cooperation with the data owner. For more information under which conditions the data can be accessed, or regarding code availability, please contact the authors.
