# Supplementary material for: Obesity as pleiotropic risk state for metabolic and mental health throughout life
Source: Transl Psychiatry. 2023 May 30;13:175. doi: 10.1038/s41398-023-02447-w (PMC10227059; doi:10.1038/s41398-023-02447-w)
Supplement: Supplementary file 1 — Supplemental Material [file 41398_2023_2447_MOESM1_ESM.docx]

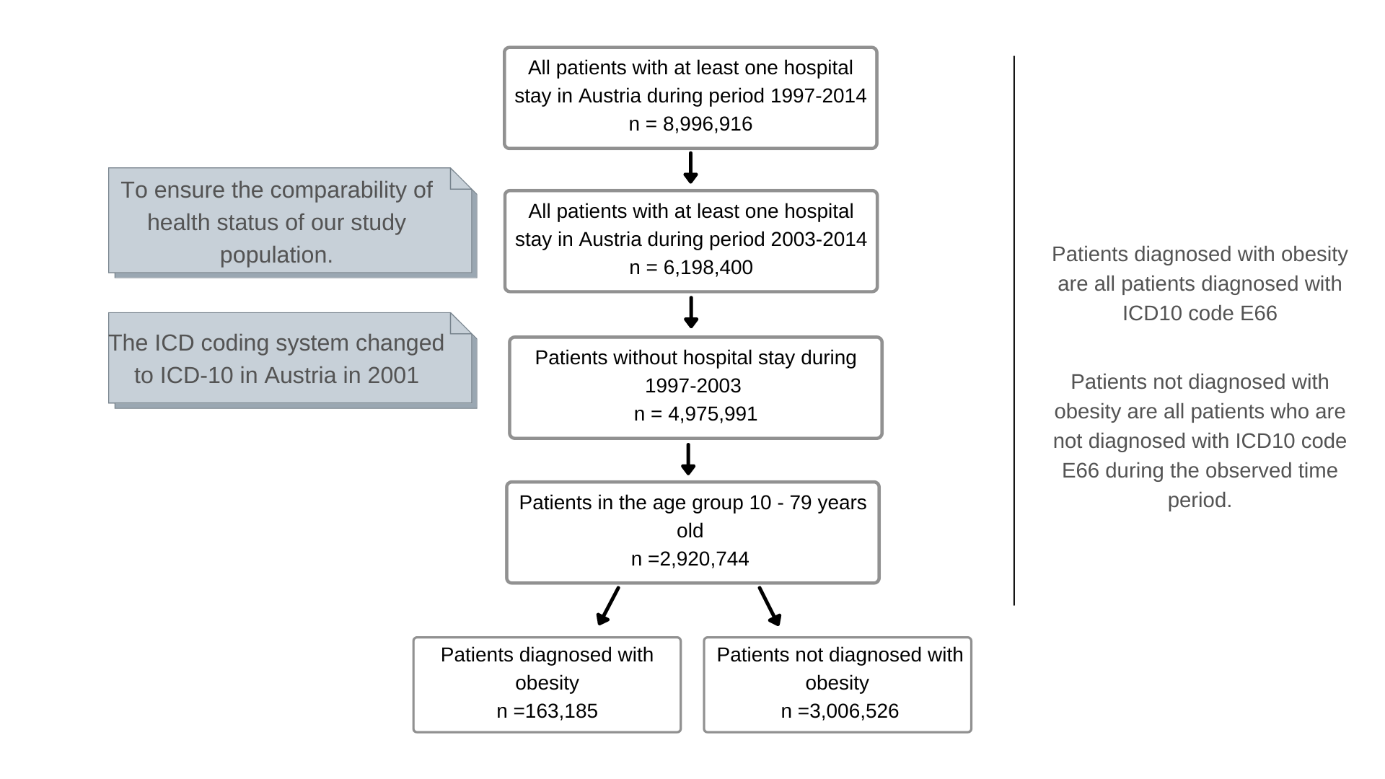


Supplementary Figure 1. Flow chart of the selection process of the analyzed study cohort.


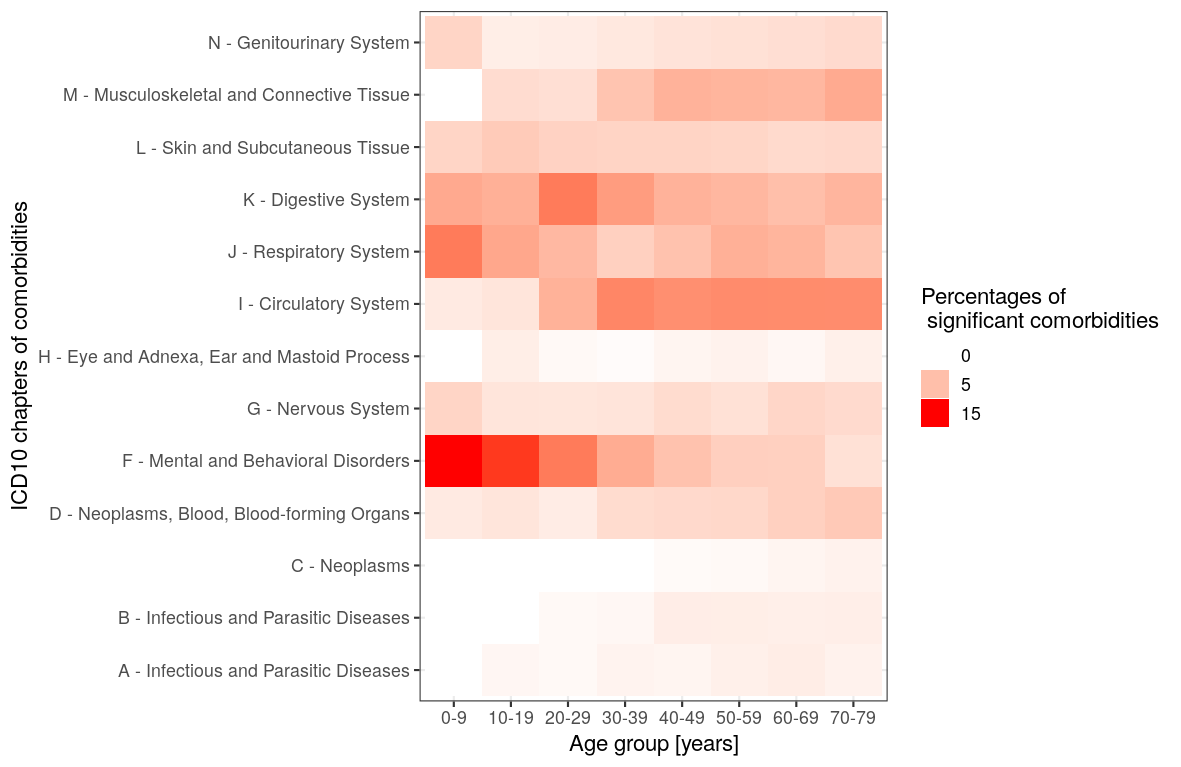
**Supplementary Figure 2.** Heat map of percentages of significant comorbidities between obesity (ICD-10: E66) and other disorders grouped by ICD-10 chapters A to N, stratified by age groups.
